# Increased aerosols can reverse Twomey effect in water clouds through radiative pathway

**DOI:** 10.1038/s41598-022-25241-y

**Published:** 2022-11-30

**Authors:** Pradeep Khatri, Tadahiro Hayasaka, Brent N. Holben, Ramesh P. Singh, Husi Letu, Sachchida N. Tripathi

**Affiliations:** 1 Center for Atmospheric and Oceanic Studies, Tohoku University, Sendai, Japan; 2 National Aeronautics and Space Administration, Goddard Space Flight Center, Greenbelt, USA; 3 School of Life and Environmental Sciences, Schmid College of Science and Technology, Chapman University, Orange, CA USA; 4 Institute of Remote Sensing and Digital Earth, Chinese Academy of Sciences, Beijing, China; 5 Department of Civil Engineering, Indian Institute of Technology Kanpur, Kanpur, India

**Keywords:** Atmospheric science, Climate change

## Abstract

Aerosols play important roles in modulations of cloud properties and hydrological cycle by decreasing the size of cloud droplets with the increase of aerosols under the condition of fixed liquid water path, which is known as the first aerosol indirect effect or Twomey-effect or microphysical effect. Using high-quality aerosol data from surface observations and statistically decoupling the influence of meteorological factors, we show that highly loaded aerosols can counter this microphysical effect through the radiative effect to result both the decrease and increase of cloud droplet size depending on liquid water path in water clouds. The radiative effect due to increased aerosols reduces the moisture content, but increases the atmospheric stability at higher altitudes, generating conditions favorable for cloud top entrainment and cloud droplet coalescence. Such radiatively driven cloud droplet coalescence process is relatively stronger in thicker clouds to counter relatively weaker microphysical effect, resulting the increase of cloud droplet size with the increase of aerosol loading; and vice-versa in thinner clouds. Overall, the study suggests the prevalence of both negative and positive relationships between cloud droplet size and aerosol loading in highly polluted regions.

## Introduction

Aerosols, tiny suspended particles in the atmosphere, are known to influence the Earth's climate system by directly scattering and absorbing solar and terrestrial radiations^1–3^ (aerosol-radiation interaction) as well as by acting as cloud condensation nuclei (CCN) and ice nuclei (IN) to modify the properties of clouds^4–7^ (aerosol-cloud interaction). These interactions show the fast response to the climate system by rapidly adjusting the temperature and moisture profiles and cloud water content^8^. As stated in the 6^th^ assessment report of the Intergovernmental Panel for Climate Change (IPCC), the estimated effective radiative forcings for aerosol-radiation and aerosol-cloud interactions are − 0.3 ± 0.3 W m^−2^ and − 1.0 ± 0.7 W m^−2^, respectively^8^. This shows that both these interactions have large uncertainties. More specifically, the uncertainty related to aerosol-cloud interaction is larger than that to aerosol-radiation interaction^9^, indicating that the level of scientific understanding for the aerosol-cloud interaction process is very low. As a result, this aerosol-cloud interaction process is poorly represented in the global climate models^9^, causing uncertainties in future climate prediction^8^.

The aerosol loading enhances CCN concentration, resulting the decrease of cloud droplet size and then the increase of cloud droplet number concentration at a fixed liquid water path (LWP). This is commonly known as the first indirect effect or Twomey effect in the literature^4–6^. In the case of thick clouds with warm cloud base and cold cloud top, the small-sized cloud droplets easily move upward and freeze at the freezing level. This releases the latent heat and invigorates the convective system, resulting in the increase of both cloud cover and cloud top height^10–12^. On the other hand, in water clouds, these small-sized cloud droplets suppress the precipitation and increase the lifetime of clouds^5,6^. Thus, modifications of cloud properties by aerosol particles are mediated through the change in cloud droplet size. Therefore, it is very important to understand aerosol associated impacts on cloud droplet size to unravel the impacts of man-made changes on the hydrological cycle and climate system. At the same time, such information greatly helps to improve future climate prediction by improving the aerosol-cloud interaction process in global climate models. However, how aerosols modify cloud droplet size remains an open question at present. There exist enough studies showing not only the decrease of cloud droplet size with the increase of aerosols (hereafter referred to as negative relationship)^4–6^, but also the increase of cloud droplet size with the increase of aerosols (hereafter referred to as positive relationship)^13–15^. The mechanism for the negative relationship is reasonably well understood since long ago^4,16^; however, there exist different arguments for the positive relationship. Qiu^17^ suggested the relative abundances of water vapor and aerosols in the atmosphere as key factors for the positive and negative relationships. Zhao^18^ suggested the important effects of water-competency and collision-coalescence efficiency among droplets for occurrences of positive and negative relationships. On the other hand, Yang^19^ pointed out that drizzle in clouds can play a decisive role for the positive and negative relationships. Additionally, it has been suggested that the excessive presence of hygroscopic aerosols and/or giant CCN can suppress super saturation and hinder the activation of smaller particles into cloud droplets, leading the positive relationship^20–22^. Some studies further showed that intense competition of water vapor due to increased aerosols can lead the evaporation of small cloud droplets, and thereby the positive relationship^14,15^. These studies provide a clue that the positive relationship can result from pathways different than the pathway of direct aerosol-cloud interaction, but these pathways are yet to be understood in detail. Here, by analyzing observation data, we discuss that cloud properties modification through the pathway of aerosol-radiation interaction can counter direct aerosol-cloud interaction process to cause both the negative and positive relationships over highly polluted regions. We further discuss the positive relationship from a new perspective by showing that cloud droplet coalescence process can be enhanced by increased aerosols to lead this phenomenon.

To generate qualitative evidences, we have had enough attentions in both data quality and study method. First, unlike the general practice of using satellite observed aerosol data^13–15^, we have used high-quality aerosol data available from AERONET^23^, a surface-based aerosol observation network, on noting that (i) the complex 3-D radiative transfer effect in the vicinity of clouds challenges aerosol observations from the space^22,24^, (ii) over land areas, where the positive relationships are often noted, the complex surface reflection function further deteriorates the quality of satellite-based aerosol products^25^, and (iii) uncertainties can be large in both small and high values of satellite-based aerosol optical thickness (AOT) due to a difficulty in observing a very pristine atmosphere^26^ and separating clouds from aerosols^27^, respectively. Furthermore, the meteorological factors are known to influence aerosol-cloud interaction process significantly^6,28^, urging it equally important to disentangle the effects of meteorological factors to understand aerosol-cloud interaction process more precisely. In view of this importance, we have followed a statistical approach to disentangle the effects of meteorological factors by treating aerosols and meteorological factors as 'stakeholders' in modification of cloud properties.

In the present study, we have used aerosol, cloud, and meteorological data corresponding to two AERONET sites (Kanpur and Gandhi College) of Indo-Gangetic Plain (IGP). Data available during the period of 2001–2019 are used. The Kanpur (26.513° N, 80.232° E) is highly polluted urban and industrial city^29^ locating ~ 400 km south-east of a mega city, New Delhi. The Gandhi College (28.871° N, 84.128° E) located in Ballia district of Uttar Pradesh is basically a rural village and is situated downwind of major urban sectors, including New Delhi, Lucknow, and Kanpur. Therefore, Kanpur is dominated by urban and industrial emissions, whereas a mixture of rural and urban aerosol emissions dominates over Gandhi College. Dust aerosols further transport over this region primarily in the pre-monsoon season^30,31^. Such diverse aerosol emissions from various sources along with dense population cause persistent heavy aerosol loading over the IGP throughout the year, making it one of the major aerosol hotspots in the world^32^. These aerosols are found to degrade air quality, and also travel over long distances impacting Himalayan glacier retreat^33,34^ and Indian summer monsoon^35,36^. Thus, understanding aerosol associated impacts on cloud properties over this region is very important to better understand the water cycle and climate system of South Asia.

## Results

**Figure 1:**
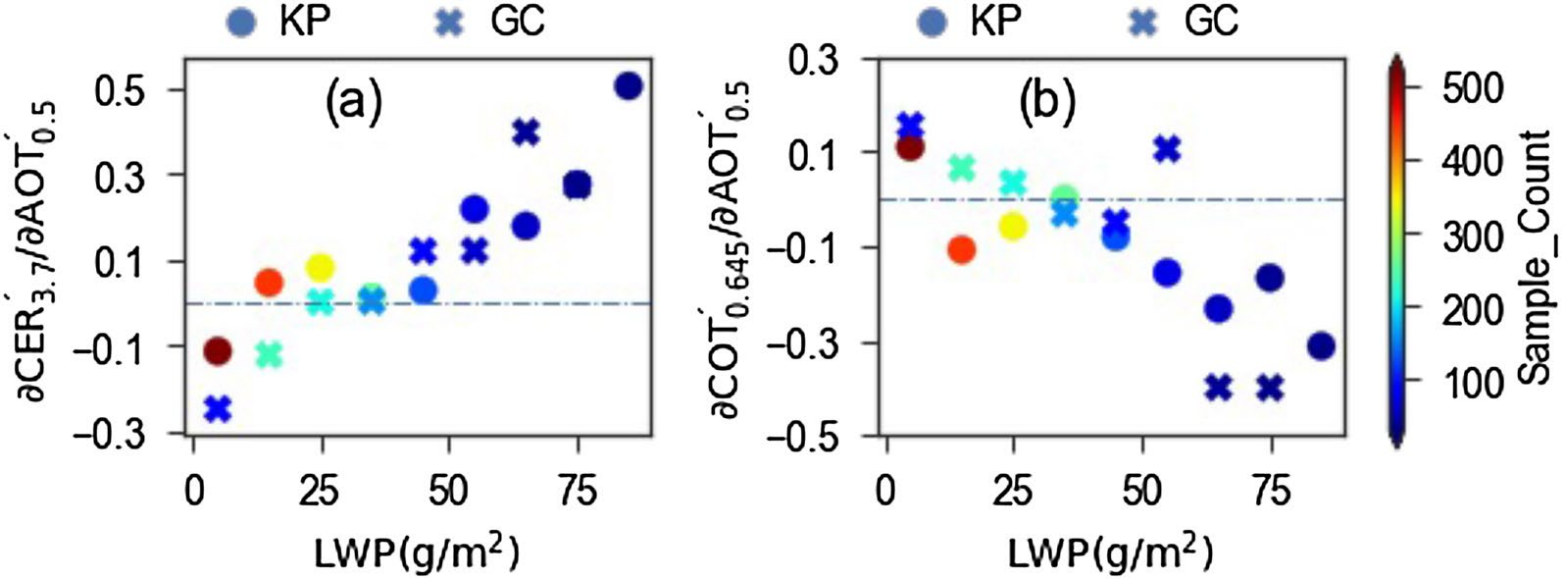
Values of (**a**) ∂${\text{CER}}_{3.7}^{^{\prime}} /\partial {\text{AOT}}_{0.5}^{^{\prime}}$ and (**b**) ∂${\text{COT}}_{0.645}^{^{\prime}} /\partial {\text{AOT}}_{0.5}^{^{\prime}}$ after decoupling the effects of meteorological factors through multiple linear regression for LWP bins of 10 g/m^2^ spectrum for Kanpur (KP) and Gandhi College (GC) sites, where ${\text{CER}}_{3.7}^{^{\prime}}$, ${\text{COT}}_{0.645}^{^{\prime}}$, and ${\text{AOT}}_{0.5}^{^{\prime}}$ are normalized anomalies of CER at 3.7 µm, COT at 0.645 µm and AOT at 0.5 µm, respectively.

AERONET provides aerosol data generated from direct sun measurements^37^ and almucantar measurements^38^. As direct sun measurement-based products have higher temporal resolution than almucantar measurement-based products, data counts are higher in the former than in the latter. Taking this advantage, aerosol data obtained from direct sun measurements are used to understand aerosol effects on cloud droplet effective radius (CER) and cloud optical thickness (COT). Aerosol and cloud data are binned for LWP with a bin width of 10 g/m^2^ to determine ∂CER´/∂AOT´ and ∂COT´/∂AOT´ for each LWP bin after decoupling the effects of meteorological factors through multiple linear regression analysis^39^ (see “Methods”). Here, x´ (x is CER, COT, or AOT) is the normalized anomaly (see “Methods”). Figure 1a,b show ∂CER´/∂AOT´ for CER corresponding to wavelength of 3.7 µm (MODIS band 20) and ∂COT´/∂AOT´ for COT corresponding to wavelength of 0.645 µm (MODIS band 1), respectively, for LWP bins having sample count greater than 30 for the regression analysis. In both of them, AERONET AOT is for 0.5 µm. The significance tests for regression models used to determine ∂${\text{CER}}_{3.7}^{^{\prime}} /\partial {\text{AOT}}_{0.5}^{^{\prime}}$ and ∂${\text{COT}}_{0.645}^{^{\prime}} /\partial {\text{AOT}}_{0.5}^{^{\prime}}$ are performed using F-test. The p-value and correlation coefficient (r) value for regression models corresponding to results shown in Fig. 1 are summarized in Table 1. As suggested by Table 1, the regression models for different LWP bins are significant with more than 99.5% confidence level (α = 0.005), except for three cases for Gandhi College site: ∂${\text{CER}}_{3.7}^{^{\prime}} /\partial {\text{AOT}}_{0.5}^{^{\prime}}$ for 0 g/m^2^ ≤ LWP < 10 g/m^2^ (p-value = 0.087), ∂${\text{COT}}_{0.645}^{^{\prime}} /\partial {\text{AOT}}_{0.5}^{^{\prime}}$ for 0 g/m^2^ ≤ LWP < 10 g/m^2^ (p-value = 0.03), and ∂${\text{COT}}_{0.645}^{^{\prime}} /\partial {\text{AOT}}_{0.5}^{^{\prime}}$ for 70 g/m^2^ ≤ LWP < 80 g/m^2^ (p-value = 0.01). Overall speaking, all results, except for ∂${\text{CER}}_{3.7}^{^{\prime}} /\partial {\text{AOT}}_{0.5}^{^{\prime}}$ for 0 g/m^2^ ≤ LWP < 10 g/m^2^, are within 95% confidence interval. Similarly, as shown in Table 1, the r value ranges from 0.21 to 0.73 in different cases. In general, the r value increases with the increase of LWP. As the results shown in Fig. 1 are derived by considering two meteorological factors—lower tropospheric stability (LTS) and precipitable water content(PWC)—in the regression model (see "Methods"), a sensitivity study is performed by increasing these meteorological factors in the regression model. In the sensitivity study, $\partial {\text{CER}}_{3.7}^{^{\prime}} /\partial {\text{AOT}}_{0.5}^{^{\prime}}$ and $\partial {\text{COT}}_{0.645}^{^{\prime}} /\partial {\text{AOT}}_{0.5}^{^{\prime}}$ are calculated for two additional sets of meteorological factors (LTS, LCL, and PWC; LTS, LCL, BLH, and PWC), where LCL and BLH are lifting condensation level and boundary layer height, respectively. The results obtained from such sensitivity study, along with those shown in Fig. 1, are shown in Supplementary Fig. S1. Supplementary Figure S1 reveals that such increase in meteorological factors has a mere influence on the estimated values of ∂${\text{CER}}_{3.7}^{^{\prime}} /\partial {\text{AOT}}_{0.5}^{^{\prime}}$ and ∂${\text{COT}}_{0.645}^{^{\prime}} /\partial {\text{AOT}}_{0.5}^{^{\prime}}$, which further justify the robustness of results shown in Fig. 1. As 3.7 µm wavelength has a weaker cloud-depth penetration compared to shorter wavelengths (e.g., 1.6, 2.1 µm) used in cloud remote sensing, this wavelength provides information of droplet size for upper cloud layers. Figure 1a suggests that the response of increased AOT on CER (3.7 µm) is different for relatively thin (low LWP) and thick (high LWP) clouds: the thin (LWP < ~ 25 g/m^2^) and thick (LWP > ~ 25 g/m^2^) clouds clearly show the negative and positive relationships, respectively. Figure 1a further reveals that the positive relationship becomes stronger with the increase of LWP. On the other hand, as shown in Supplementary Fig. S2, CER corresponding to 1.6 µm, which represents the droplet size for cloud layers deeper than that for 3.7 µm, hardly shows such increase of the strength of positive relationship with the increase of LWP. The p-value and r value for the regression models corresponding to results of Supplementary Fig. S2 are given in Supplementary Table S1. In general, aerosols near the cloud base influence the lower and/or middle cloud layers through direct aerosol-cloud interaction process, and this effect gradually expands towards the cloud top^10,12^. Such difference regarding LWP dependent strength for positive relationship between CER(3.7 µm) and CER(1.6 µm), as noted in Figs. 1a and S2, indicates that the aerosol-cloud interaction process initiated by aerosols below the cloud base can be opposed by the next process occurring near the cloud top (see "Discussion"). Further, by coinciding with Fig. 1a,b suggests the opposite response of increased aerosols on COT with respect to that on CER. For fixed LWP, such opposite response is not surprising as LWP can be approximated as k × COT × CER, where k is a constant term depending on the vertical inhomogeneity of cloud layers^40,41^. In other words, decreased (increased) CER can increase (decrease) cloud droplet concentration to increase (decrease) total cross section area and COT when LWP remains unchanged. Therefore, the positive relationship is associated with not only CER increment, but also COT decrement. Though a large volume of studies discussed CER increment with the increase of aerosols; however, to the best of our knowledge, this consequent response of increased aerosols on COT decrement has not been discussed in detail. The most plausible explanation for such COT decrement along with CER increment is cloud droplet coalescence process. This suggests that cloud droplet coalescence process can cause the positive relationship between CER and aerosol loading.

**Table 1:** Results of p-value and correlation coefficient (r) value for regression models used to determine ∂${\text{CER}}_{3.7}^{^{\prime}} /\partial {\text{AOT}}_{0.5}^{^{\prime}}$ and ∂${\text{COT}}_{0.645}^{^{\prime}} /\partial {\text{AOT}}_{0.5}^{^{\prime}}$ for Kanpur (KP) and Gandhi College (GC) shown in Fig. 1.

LWP bin (g/m^2^)	Kanpur (KP)				Gandhi College (GC)			
	∂${\text{CER}}_{3.7}^{^{\prime}} /\partial {\text{AOT}}_{0.5}^{^{\prime}}$		${\text{COT}}_{0.645}^{^{\prime}} /\partial {\text{AOT}}_{0.5}^{^{\prime}}$		∂${\text{CER}}_{3.7}^{^{\prime}} /\partial {\text{AOT}}_{0.5}^{^{\prime}}$		${\text{COT}}_{0.645}^{^{\prime}} /\partial {\text{AOT}}_{0.5}^{^{\prime}}$	
	p-value	r	p-value	r	p-value	r	p-value	r
0 ≤ LWP < 10	2.10E−05	0.24	3.44E−29	0.48	8.27E−02	0.32	2.96E−02	0.32
10 ≤ LWP < 20	3.24E−04	0.21	4.04E−23	0.46	3.29E−04	0.28	1.03E−07	0.37
20 ≤ LWP < 30	4.60E−19	0.48	3.34E−35	0.62	2.19E−06	0.36	1.94E−13	0.51
30 ≤ LWP < 40	1.03E−16	0.51	1.22E−25	0.61	1.62E−11	0.54	3.21E−11	0.53
40 ≤ LWP < 50	7.58E−13	0.61	1.10E−09	0.55	3.56E−10	0.64	2.48E−06	0.52
50 ≤ LWP < 60	3.48E−05	0.53	9.19E−04	0.45	1.64E−06	0.62	2.13E−04	0.52
60 ≤ LWP < 70	2.77E−03	0.47	1.31E−04	0.55	6.03E−03	0.54	4.94E−06	0.72
70 ≤ LWP < 80	2.41E−04	0.64	9.01E−06	0.72	1.03E−03	0.63	1.10E−02	0.55
80 ≤ LWP < 90	2.64E−05	0.73	8.31E−05	0.70				

**Figure 2:**
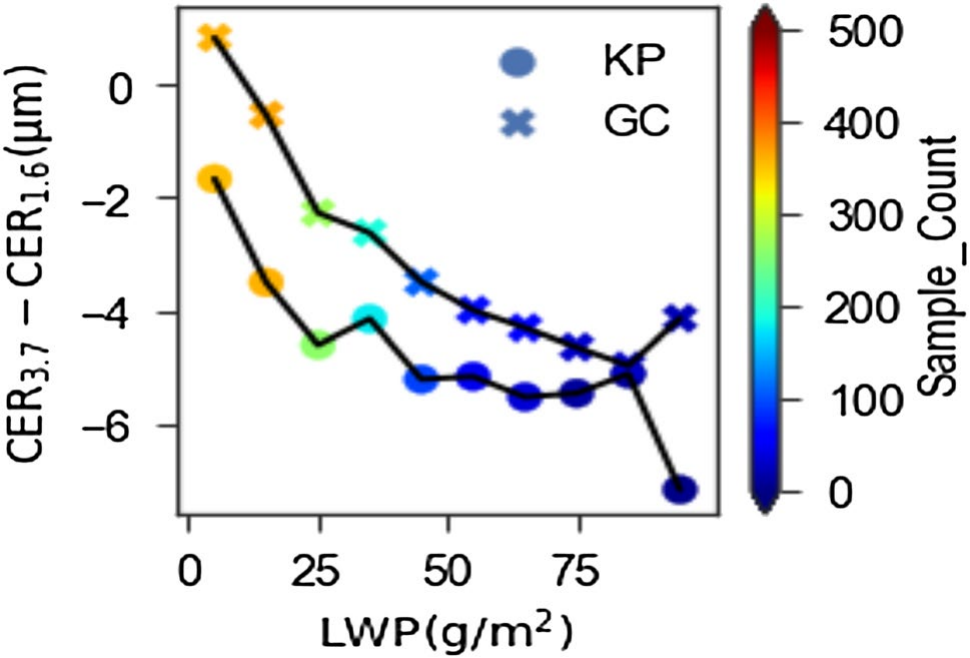
Differences between CERs at 3.7 and 1.6 µm for LWP bins of 10 g/m^2^ spectrum for Kanpur (KP) and Gandhi College (GC) sites.

In order to get better insight into such cloud droplet coalescence process over our study regions, we analyzed the difference in CER between 3.7 µm and 1.6 µm (CER_3.7_-CER_1.6_). It is because CER corresponding to 3.7 µm becomes smaller than that for 2.1 µm or 1.6 µm wavelength, if coalescence growth process dominates over condensation growth process, and vice-versa^42,43^. As shown in Fig. 2, the mean values of CER_3.7_-CER_1.6_ are negative for all LWP bins, suggesting the dominance of coalescence growth process over both study regions. Figure 2 further shows that the CER_3.7_-CER_1.6_ becomes more negative with the increase of LWP. This suggests that coalescence growth process becomes stronger in thicker clouds than in thinner clouds. It is because thicker clouds provide sufficient time and opportunity than thinner clouds for cloud droplets to collide. This behavior is important for the occurrences of negative and positive relationships in relatively thinner and thicker clouds, respectively, as noted in Fig. 1. It is because the microphysical effect, which is primarily initiated from the cloud base, is an indispensable part regardless of the presence or absence of the radiative effect that occurs when the microphysical effect becomes saturated (see "Discussion"). As aerosols near the cloud base can easily interact with the middle layers or even higher layers in thinner clouds than in thicker clouds, the microphysical effect can strongly modify cloud properties in thinner clouds than in thicker clouds. Since the coalescence growth process is weaker in thinner clouds, the strong microphysical effect that occurs before the saturation point is weakly opposed in thinner clouds, resulting the net effect dominated by the beforehand microphysical effect. On the other hand, stronger coalescence growth process can overcome weaker microphysical effect in thicker clouds, resulting the net effect dominated by coalescence growth process. This phenomenon plausibly describes the negative and positive relationships for relatively thinner and thicker clouds, respectively, over highly aerosol loaded regions, such as IGP. Overall speaking, prevalence of negative and positive relationships is the outcome of the competition between the microphysical and radiative effects.

Aerosol Index (AI), a product of AOT and Ångström exponent (AE), is also used as a proxy of aerosol number concentration (N_tot_) while quantifying aerosol effects on cloud properties^5,13,14,28^, assuming that AI can represent N_tot_ better than AOD^5^. AE is estimated either by paring AOTs of two wavelengths or through a least square fit of AOTs observed at discrete wavelengths. Therefore, depending on the choice of wavelengths, the calculated AE can differ^44,45^ to affect AI as well, and thereby quantification of aerosol led impacts on cloud properties. Supplementary Fig. S3 shows ∂CER´/∂AI´ for AIs corresponding to AEs of different wavelength combinations made public by AERONET (denoted as AE_0.34–0.44_ for combinations of 0.34, 0.38, and 0.44; and similar for combinations of 0.44, 0.5, and 0.675 µm; and 0.5, 0.675, and 0.87 µm) and calculated in this study for the combination of 0.44, 0.5, 0.67, 0.87, and 1.02 µm wavelengths. The p-value and r value for the regression models corresponding to results shown in Fig. S3 are given in Supplementary Table S2. Figure S3 confirms that quantified aerosol effects on cloud properties are subject to change depending on the choice of wavelengths for AE calculation. It is because AE is related to aerosol size distribution^46^; and fine-mode and coarse-mode aerosols exhibiting larger sensitivities to the longer and shorter wavelengths, respectively, are differently weighted depending on wavelengths in AE calculation^47^. Figure S3 shows that such wavelength choice for AE (and AI) calculation can affect the quantified values for both thinner and thicker clouds; however, relatively, thinner clouds may be affected more prominently than thicker clouds. Among different wavelength combinations, the one with relatively wider spectral range (0.44–1.02 µm) shows results closer to Fig. 1a, which use AOT as a proxy of aerosol concentration. This suggests that AI estimated from AE of relatively wider spectral range may better represent aerosol loading, as such wider spectral range can more reasonably weight fine- and coarse-mode contributions in aerosol size distribution than narrow spectral range. Taking an opportunity of aerosol size distribution data (dV/dlnr) available in almucantar measurements^38^, we further tested if AI is indeed a good proxy for N_tot_. For this purpose, the calculated N_tot_ values from aerosol size distributions (see "Methods") are compared with both AOT (0.5 µm) and AI separately, as shown in Supplementary Fig. S4. Figure S4 shows that the varying AE can largely deteriorate the relationship between AI and N_tot_, suggesting that AI may not be a better choice to consider a proxy of N_tot_ over regions of varying AE (or aerosol type). On the other hand, the improved agreement for AOT and N_tot_ relationship suggests AOT, rather than AI, better represents N_tot_ over such regions. Therefore, AOT can better quantify aerosol led impacts on cloud properties over the study regions of this study.

**Figure 3:**
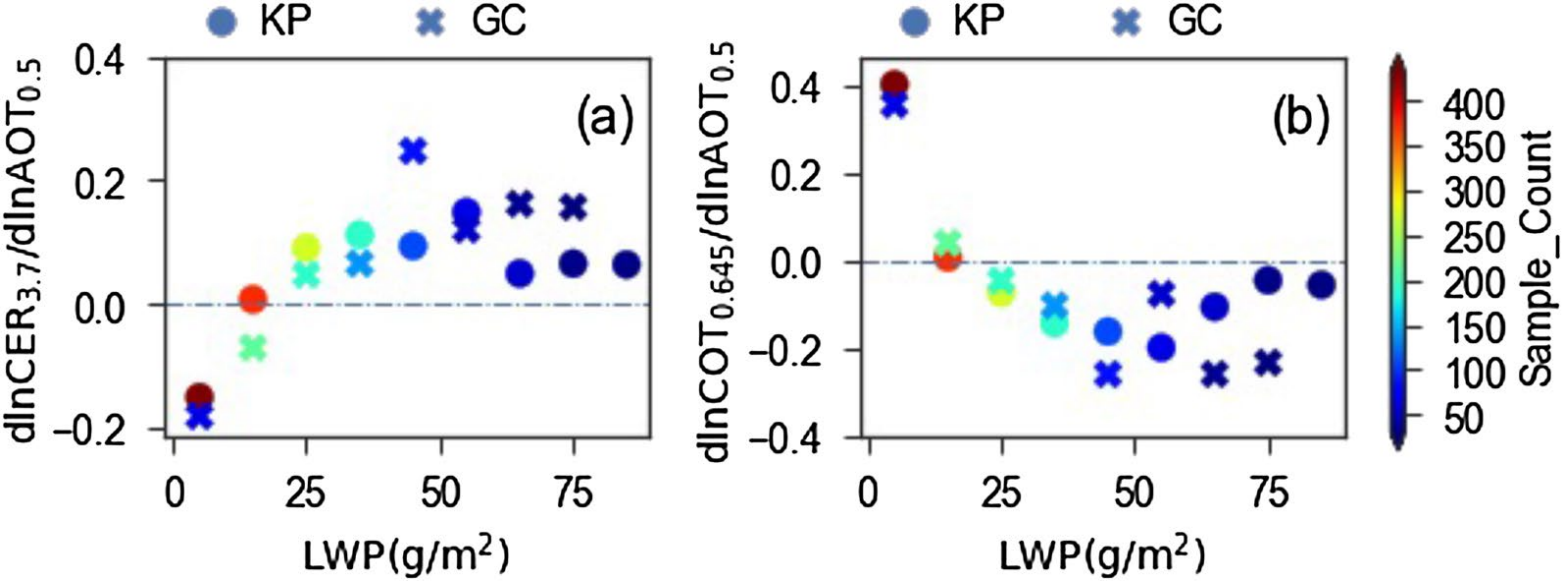
Values of (**a**) dlnCER_3.7_/dlnAOT_0.5_ and (**b**) dlnCOT_0.645_/dlnAOT_0.5_ for LWP bins of 10 g/m^2^ spectrum for Kanpur (KP) and Gandhi College (GC) sites, where CER_3.7_, COT_0.645_, and AOT_0.5_ are CER at 3.7 µm, COT at 0.645 µm and AOT at 0.5 µm, respectively.

The aerosol led impacts on cloud properties are generally quantified in the form of aerosol indirect effect (AIE) as dlnCLD/dlnALD^13,14,21^, where CLD is CER or COT, and ALD is AOT or AI. We further followed this approach to quantify AIEs for each LWP bin. The quantified values corresponding to CER of 3.7 µm and COT of 0.645 µm are shown in Fig. 3a,b, respectively. Both Fig. 3a,b show qualitative agreement with Fig. 1a,b, respectively, by suggesting the negative and positive relationships for relatively thinner (low LWP) and thicker (high LWP) clouds, respectively. However, Fig. 3 does not show the increase in the strength of positive relationship with the increase of LWP very clearly, such as those observed in Fig. 1. This could be due to the influence of meteorological factors on cloud properties^6,28^, as such influences are left untouched in this approach. Despite it, Fig. 3 supports the robustness for negative and positive relationships for relatively thinner and thicker clouds, respectively, over our study regions, as shown in Fig. 1. At the same time, by highlighting the influences of meteorological factors on AIE, Fig. 3 suggests it important to disentangle these meteorological influences to better understand aerosol led impacts on cloud properties.

## Discussion

**Figure 4:**
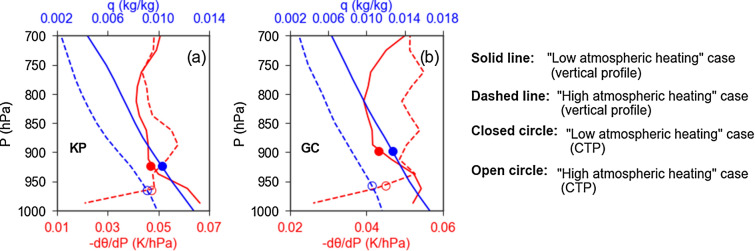
Vertical profiles of specific humidity (q) and potential temperature gradient (-dθ/dp) along with CTP for "low atmospheric heating" and "high atmospheric heating" cases for (**a**) Kanpur (KP) and (**b**) Gandhi College (GC) sites.

**Figure 5:**
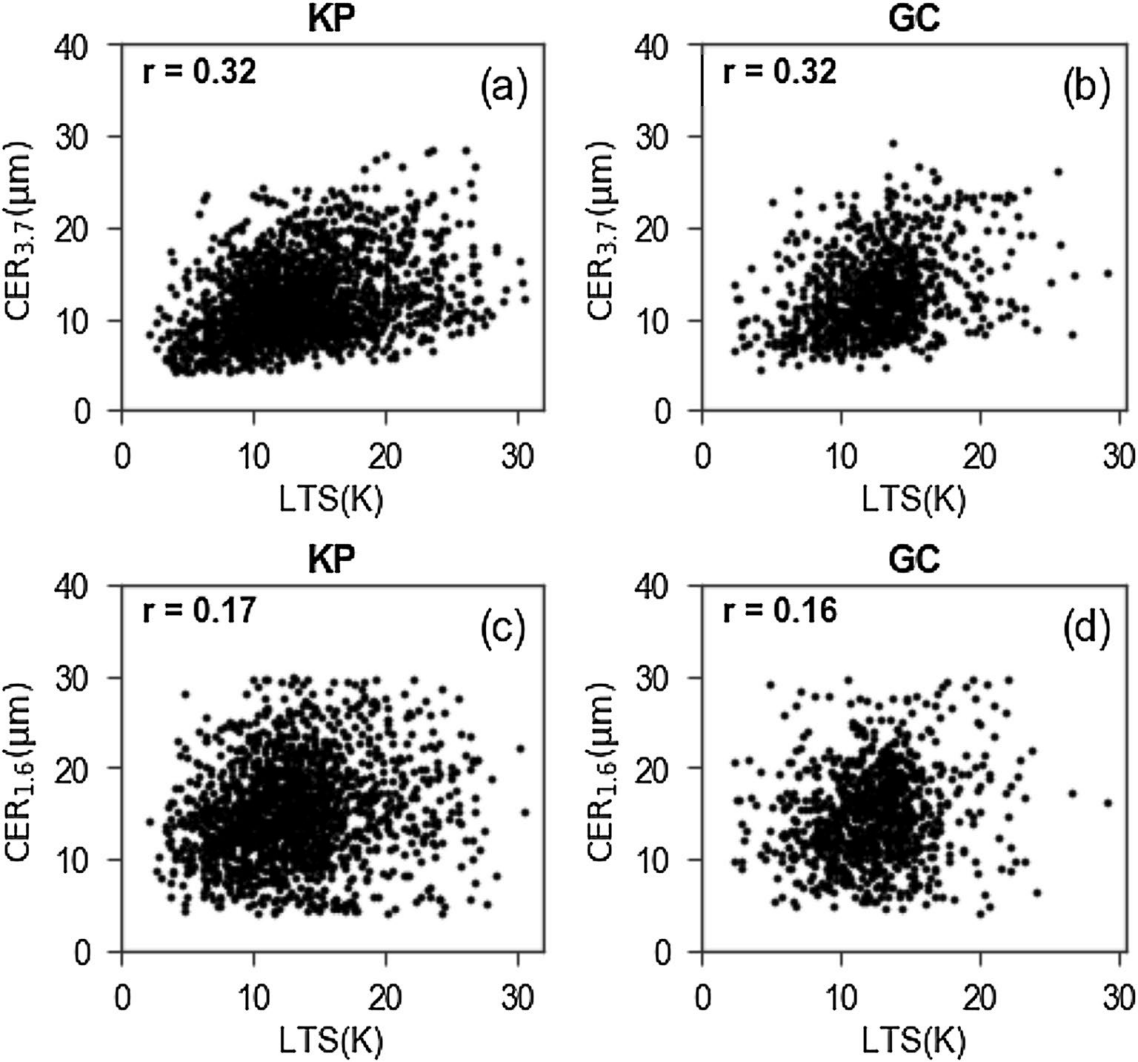
Correlations between CER at 3.7 µm and LST for (**a**) Kanpur (KP) and (**b**) Gandhi College (GC) sites. Same in (**c**) and (**d**) for CER at 1.6 µm.

There are two pathways for aerosols to influence cloud properties: microphysical and radiative^48,49^. In the former, aerosols directly interact with clouds by acting as CCN. But, in the latter, aerosols play an important role by absorbing^21,48^ and/or scattering^50^ the solar radiation and then altering the meteorological conditions, e.g., atmospheric stability and moisture content. The microphysical effect increases logarithmically with the increase of AOT until reaching a saturation point^48^. Though it depends on the meteorological condition, the microphysical effect generally saturates when AOT at 0.5 µm increases up to ~ 0.3 or more^21,48^. After saturation, the radiative effect gradually overrides the former microphysical effect^48^. Thus, the radiative pathway can play a critical role to modulate cloud properties over highly polluted regions with AOT (0.5 µm) exceeding ~ 0.3. Supplementary Fig. S5 shows the frequency distributions of AOT (0.5 µm), corresponding to the direct sun measurement, and single scattering albedo (SSA) at 0.44 µm, corresponding to the almucantar measurement, for both study regions. The peak value of AOT (0.5 µm) falls above 0.3 over both study regions, suggesting the prevalence of high aerosol loadings over both study regions. Furthermore, the peak value of SSA (0.44 µm) is less than 0.95 over both study regions, suggesting that these highly loaded aerosols are quite light absorbing as well. Overall, aerosols of both study regions are highly capable to trap (absorb) solar radiation within the atmosphere with potential of modulating cloud properties through the radiative pathway mentioned above. Supplementary Fig. S6 shows the scatterplot for aerosol loading and the amount of solar energy trapped within the atmosphere, i.e., atmospheric forcing (ATM), which is the difference in aerosol radiative forcing between the top of the atmosphere (TOA) and the surface, over our study regions. These calculated ATMs and collocated meteorological data (see "Data") are used to illustrate how aerosol led atmospheric heating via solar radiation absorption can modulate the vertical distributions of atmospheric stability and moisture content. For this purpose, the calculated ATMs are sorted in an ascending order and binned into three segments of equal sample numbers. Then, the potential temperature (θ) gradient (-dθ/dp) and specific humidity (q) profiles of pressure (p) coordinated atmospheric layers corresponding to data of the first segment ("low atmospheric heating" case) and the third segment ("high atmospheric heating" case) of sorted ATMs are averaged. The mean values of ATM corresponding to the first and third segments are 26.9 ± 8.7 Wm^−2^ (28.4 ± 8.9 Wm^−2^) and 82.5 ± 20.2 Wm^−2^ (84.6 ± 20.3 Wm^−2^), respectively, for Kanpur (Gandhi College). Note that ATM can be converted into atmospheric heating rate as k' × ATM/ΔP, where k' is a constant term and ΔP is the pressure difference between the surface and TOA^51,52^. Along with the mean values of cloud top pressure (CTP), the vertical profiles of q (blue lines) and − dθ/dp (red lines) for "low atmospheric heating" case (solid lines) and "high atmospheric heating" case (dashed lines) are shown in Fig. 4. The positive value of − dθ/dp suggests a stable atmospheric layer, and vice-versa. Figure 4 suggests that the increased atmospheric heating is associated with the decrease of moisture content within the atmosphere. Since both study regions have high AOTs with relatively low SSAs, aerosols of these regions can have important contributions to heat the atmosphere and then to suppress the moisture flux from the surface by cooling the surface as well as by reducing the surface evaporation due the decrease of solar radiation at the surface^48^. Such decrease of moisture content in the atmosphere can increase the competition for water vapor, leading the evaporation of smaller cloud droplets, and thereby the positive relationship^14,15,21^. Figure 4 further suggests higher values of -dθ/dp at altitudes higher than cloud top heights in "high atmospheric heating" case compared to "low atmospheric heating" case. This suggests that atmospheric stability at higher altitudes can be enhanced by increased aerosols via radiative pathway. Such increased stability at such higher altitudes plays an important role to affect cloud properties near the cloud top, as revealed from Fig. 5. Figure 5 shows the correlations of LTS, the difference in θ between 700 and 1000 hPa, with CER (3.7 µm) and CER (1.6 µm). Over both study regions, LTS correlated strongly with CER (3.7 µm) than with CER (1.6 µm), indicating the important role of such increased stability on modulating the properties of clouds near the cloud top. Further, as stable air mass has a tendency to sink, enhanced atmospheric stability at such higher altitudes can amplify the entrainment mixing process near the cloud top^53^. The entrainment mixing process can also play an important role to decrease the cloud top height. As a result, Fig. 4 suggests the decrease of cloud top height in "high atmospheric heating" case compared to "low atmospheric heating" case. It is important to note that the entrained air causes both dilution and evaporation of cloud droplets, helping to broaden cloud droplet spectra^54–56^. As larger cloud droplets have higher terminal velocities, they fall faster and collide with smaller cloud droplets. The aggregated droplets fall even faster to collide and coalescence with smaller droplets in their path. This process becomes more efficient for wider distribution of cloud droplet size, as terminal velocity depends on cloud droplet size. Therefore, cloud droplet spectra broadened by entrainment mixing process generates a better condition for cloud droplet coalescence process. Thus, increased aerosols can contribute in cloud droplet coalescence process by increasing atmospheric stability at higher altitudes over both study regions. A modelling study has also shown the increase (decrease) of CER (cloud droplet concentration) through entrainment process in polluted clouds^53^ to support the results of this study.

## Data

### AERONET

Level 2.0 (Version 3.0) data of aerosol optical thickness (AOT) at 0.5 µm and precipitable water content (PWC) available from direct sun measurement^37^; AOT, single scattering albedo (SSA), and asymmetry parameter (ASY) at the wavelengths of 0.44, 0.67, 0.87, and 1.02 µm available from almucantar measruements^38^ are used. Level 2.0 SSAs are available only for AOT (0.44 µm) greater than 0.4. AERONET further provides information of surface reflectance at the wavelengths of 0.44, 0.67, 0.87, and 1.02 µm by combining down and up-looking observations from surface and space^57^, which are also used. Level 2.0 data available for the periods of 2001–2019 and 2006–2019 for Kanpur (26.513° N, 80.232° E) and Gandhi College (28.871° N, 84.128° E), respectively, are used.

### MODIS

Level 2.0 (Collection 6.1) cloud optical thickness (COT), cloud particle effective radius, cloud liquid water path (LWP), cloud top temperature (CTT), and cloud top pressure (CTP) data of daytime observed by Moderate Resolution Spectroradiometer (MODIS) aboard Terra (equator overpass time: ~ 10:30 LT) and Aqua (equator overpass time: ~ 13:30 LT) satellites^58^ are used. COT corresponding to 0.645 µm and two sets of CER corresponding to 1.6 and 3.7 µm are used. All these data products have spatial resolution of 1 km × 1 km (at nadir). Only water clouds with CTT > 273.15 K for periods coinciding with AERONET data are used.

### ERA5

The air temperature and specific humidity of pressure coordinated atmospheric layers between 1000 hPa and 700 hP and boundary layer height (BLH) data are used. Pressure coordinated data are available for interval of 25 hPa from 1000 to 750 hPa and the interval of 50 hPa from 750 to 700 hPa. The spatial and temporal resolutions are 0.25° × 0.25° and 1-h, respectively. Data for periods coinciding with AERONET data are used.

## Study method

### Data preparation

The cloud properties of 25 km × 25 km region that centers AERONET observation sites were averaged to use in this study. Aerosol data of ± 30-min centering the time ahead of cloud observation time by 3-h were averaged. This time lag of 3-h reasonably captures the time period required for aerosol-cloud interaction^28^ by further helping to accumulate more reasonable data of aerosols and clouds for aerosol-cloud interaction study, as aerosols beneath the cloud is difficult to observe during cloudy sky condition. The hourly metrological data of each pressure level were converted into time resolution of 1-min through cubic spline interpolation. Then, metrological data of ± 30-min centering the time ahead of cloud observation time by 3-h were averaged. We used air temperature (T), pressure (P), and specific humidity (q) data in this study.

The potential temperature (θ) was calculated as

1 $$\theta =T{\left({P}_{0}/P\right)}^\frac{R}{Cp},$$

where P_0_ is pressure at 1000 hPa, R (= 287.05 Jkg^−1^ K^−1^) is the gas constant of air, and C_p_ (= 1004 Jkg^−1^ K^−1^) is the specific heat capacity at constant pressure. The potential temperature gradient for any atmospheric layer i was calculated as

2 $$-\frac{d\theta }{dP}=\frac{{\theta }_{i}-{\theta }_{i+1}}{{P}_{i+1}-{P}_{i}}.$$

The lower tropospheric stability (LTS) was calculated as

3 $$LTS = {\theta }_{700 hPa}- {\theta }_{1000 hPa}.$$

Similarly, lifting condensation level (LCL), the height at which the relative humidity of air parcel becomes saturated, is calculated from vertical profiles of P and q using Metpy Python.

### Calculation of aerosol radiative effect

In order to calculate the amount of solar flux trapped within the atmosphere due to aerosol absorption, we used AERONET observed spectral values of AOT, SSA, ASY, and surface reflectance at 0.44, 0.67, 0.87, and 1.02 µm wavelengths, and PWC. Using those data in a Santa Barba DISORT Atmospheric Radiative Transfer (SBDART) model^59^, downwelling and upwelling global radiative fluxes (spectral range: 0.3–3.0 µm) at the surface and top of the atmosphere (TOA) were calculated for aerosol laden atmosphere. Further, by assuming no aerosols in the atmosphere, the same fluxes corresponding to aerosol free atmosphere were calculated. Aerosol radiative forcing (ARF) values at the surface and TOA were calculated as

4 $${ARF}_{sfc}=\left({F}_{al,sfc}^{\downarrow }-{F}_{al,sfc}^{\uparrow }\right)-\left({F}_{af,sfc}^{\downarrow }-{F}_{af,sfc}^{\uparrow }\right),$$

5 $${ARF}_{toa}=({F}_{al,toa}^{\downarrow }-{F}_{al,toa}^{\uparrow })-{(F}_{af,toa}^{\downarrow }-{F}_{af,toa}^{\uparrow }),$$

where, the subscripts sfc, toa, al, and af denote surface, TOA, aerosol laden and aerosol free, respectively. Finally, the amount of solar flux trapped within the atmosphere due to aerosol absorption, i.e., atmospheric forcing (ATM), was calculated as

6 $$ATM = {ARF}_{toa} - {ARF}_{sfc}$$

### Calculation of aerosol number concentration

The aerosol volume size distribution (dV/dlnr) of 22 central radii available from the almucantar measurements of AERONET were integrated, as below, to calculate total aerosol number concentration (N_tot_).

7 $${N}_{tot}=\frac{3}{4\pi }{\int }_{{r}_{min}}^{{r}_{max}}\frac{1}{r^3}\ \frac{dV}{dlnr}dlnr$$

where r_min_ and r_max_ are the minimum and maximum values of central radii for the integration. The values of r_min_ and r_max_ can be calculated as

8 $$ln{r}_{min}=ln{r}_{1}-dlnr/2,$$

9 $$ln{r}_{max}=ln{r}_{22}+dlnr/2$$

where r_1_ and r_22_ are central radii of the 1st and 22nd bins, respectively.

### Multiple linear regression analysis

In order to quantify aerosol effects on cloud property (COT or CER) by decoupling the effect of meteorological parameters, a multiple linear regression method was applied by treating a cloud property as an independent variable and AOT and meteorological parameters as dependent variables. We formulated their relationship as

10 $$CLD^{^{\prime}} = aAOT^{^{\prime}} + \mathop \sum \limits_{i = 0}^{n} b_{i} M_{i}^{^{\prime}} ,$$

where,

11 $$CLD^{^{\prime}} = \frac{{CLD - \overline{CLD} }}{{\sigma_{CLD} }},$$

12 $$AOT^{^{\prime}} = \frac{{AOT - \overline{AOT} }}{{\sigma_{AOT} }},$$

and

13 $$M_{i}^{^{\prime}} = \frac{{M - \overline{M}}}{{\sigma_{M} }},$$

In these equations, CLD is a cloud property (COT or CER) and M_i_ is any specific meteorological parameter (e.g., PWC). Further, *bar* and *σ* represent the mean and standard deviation values, respectively. In Eq. (10), *a* and *b*_*i*_ are constant terms. We derive *a* by considering two meteorological factors, LST and PWC. The constant term a is ∂CER´/∂AOT´ (∂COT´/∂AOT´) when CLD is CER (COT).

## Supplementary Information

Supplementary Information.

## Data Availability

AERONET, MODIS, and ERA5 data used in this study are downloaded from https://aeronet.gsfc.nasa.gov, https://search.earthdata.nasa.gov/search and https://cds.climate.copernicus.eu/#!/search?text=ERA5&type=dataset, respectively.

## Code availability

Data analyses codes are available upon request to the corresponding author.
