# Author Correction: Increased aerosols can reverse Twomey effect in water clouds through radiative pathway

**DOI:** 10.1038/s41598-023-28028-x

**Published:** 2023-01-16

**Authors:** Pradeep Khatri, Tadahiro Hayasaka, Brent N. Holben, Ramesh P. Singh, Husi Letu, Sachchida N. Tripathi

**Affiliations:** 1 Center for Atmospheric and Oceanic Studies, Tohoku University, Sendai, Japan; 2 National Aeronautics and Space Administration, Goddard Space Flight Center, Greenbelt, USA; 3 School of Life and Environmental Sciences, Schmid College of Science and Technology, Chapman University, Orange, CA USA; 4 Institute of Remote Sensing and Digital Earth, Chinese Academy of Sciences, Beijing, China; 5 Department of Civil Engineering, Indian Institute of Technology Kanpur, Kanpur, India

Correction to: *Scientific Reports* 10.1038/s41598-022-25241-y, published online 30 November 2022

The original version of this Article contained errors in Table 1, where the city ‘Kanpur’ was incorrectly given as ‘Kantipur’, and in Equation 7, where the aerosol volume size distribution was not multiplied by $\frac{1}{{r}^{3}}$.

Incorrect:

$$ N_{tot} = \frac{3}{4\pi }\mathop \int \limits_{{r_{min} }}^{{r_{max} }} \frac{dV}{{dlnr}}dlnr $$

Correct:

$$ N_{tot} = \frac{3}{4\pi }\mathop \int \limits_{{r_{min} }}^{{r_{max} }} \frac{1}{{r^{3} }}\frac{dV}{{dlnr}}dlnr $$

The original Article has been corrected.

