# Supplementary material for: Increased aerosols can reverse Twomey effect in water clouds through radiative pathway
Source: Sci Rep. 2022 Nov 30;12:20666. doi: 10.1038/s41598-022-25241-y (PMC9712532; doi:10.1038/s41598-022-25241-y)
Supplement: Supplementary file 1 — Supplementary Information. [file 41598_2022_25241_MOESM1_ESM.docx]

Increased aerosols can reverse Twomey effect in water clouds through radiative pathway

Pradeep Khatri^1*^, Tadahiro Hayasaka^1^, Brent N. Holben^2^, Ramesh P. Singh^3^, Husi Letu^4^, and Sachchida N. Tripathi^5^

^1^ Center for Atmospheric and Oceanic Studies, Tohoku University, Sendai, Japan,

^2^ National Aeronautics and Space Administration, Goddard Space Flight Center, Greenbelt, USA

^3^ School of Life and Environmental Sciences, Schmid College of Science and Technology, Chapman University, Orange, CA, USA

^4^ Institute of Remote Sensing and Digital Earth, Chinese Academy of Sciences, Beijing, China

^5^ Department of Civil Engineering, Indian Institute of Technology Kanpur, Kanpur, India

* Correspondence: pradeep.khatri.a3@tohoku.ac.jp

**Supplementary tables**

**Table S1**. Results of p-value and correlation coefficient (r) value for regression models used to determine ∂$\mathrm{CER}_{1.6}^{´}/{\partial AOT}_{0.5}^{´}$ for Kanpur (KP) and Gandhi College (GC) shown in Figure S2.

| LWP bin (g/m^2^) | Kanpur (KP) | | Gandhi College (GC) | |
| --- | --- | --- | --- | --- |
|  | p-value | r | p-value | r |
| 0 ≤ LWP < 10 | 1.13E-02 | 0.16 | 5.84E-03 | 0.42 |
| 10 ≤ LWP < 20 | 4.63E-06 | 0.27 | 7.28E-02 | 0.18 |
| 20 ≤ LWP < 30 | 3.34E-11 | 0.42 | 8.33E-08 | 0.41 |
| 30 ≤ LWP < 40 | 1.08E-09 | 0.46 | 1.14E-04 | 0.38 |
| 40 ≤ LWP < 50 | 4.75E-07 | 0.51 | 2.18E-04 | 0.46 |
| 50 ≤ LWP < 60 | 1.25E-04 | 0.52 | 5.30E-02 | 0.36 |
| 60 ≤ LWP < 70 | 1.11E-02 | 0.43 | 3.70E-04 | 0.64 |
| 70 ≤ LWP < 80 | 5.32E-02 | 0.47 | 1.01E-03 | 0.66 |
| 80 ≤ LWP < 90 | 2.47E-04 | 0.69 |  |  |

**Table S2**. Results of p-value and correlation coefficient (r) value for regression models used to determine ∂$\mathrm{CER}_{3.7}^{´}/{\partial AI}^{´}$ for AI values determined from different wavelength combinations for Kanpur (KP) and Gandhi College (GC) $shown in Figure S3$.

| LWP bin (g/m^2^) | Kanpur (KP) | | | | | | | Gandhi College (GC) | | | | | | | | |
| --- | --- | --- | --- | --- | --- | --- | --- | --- | --- | --- | --- | --- | --- | --- | --- | --- |
|  | 0.34-0.44 µm | | 0.44-0.675 µm | | 0.5-0.87 µm | | 0.44-1.02 µm | | 0.34-0.44 µm | | 0.44-0.675 µm | | 0.5-0.87 µm | | 0.44-1.02 µm | |
|  | p-value | r | p-value | r | p-value | r | p-value | r | p-value | r | p-value | r | p-value | r | p-value | r |
| 0 ≤ LWP < 10 | 2.42E-04 | 0.21 | 2.54E-04 | 0.21 | 2.54E-04 | 0.21 | 7.14E-06 | 0.25 | 2.19E-01 | 0.26 | 2.07E-01 | 0.27 | 2.07E-01 | 0.27 | 1.63E-01 | 0.28 |
| 10 ≤ LWP < 20 | 3.70E-04 | 0.20 | 1.95E-04 | 0.21 | 1.95E-04 | 0.21 | 3.17E-04 | 0.21 | 1.39E-03 | 0.26 | 1.19E-03 | 0.26 | 1.19E-03 | 0.26 | 4.77E-04 | 0.28 |
| 20 ≤ LWP < 30 | 2.75E-19 | 0.48 | 6.17E-21 | 0.50 | 6.17E-21 | 0.50 | 1.89E-18 | 0.47 | 2.09E-06 | 0.36 | 2.13E-06 | 0.36 | 2.13E-06 | 0.36 | 1.60E-06 | 0.36 |
| 30 ≤ LWP < 40 | 1.03E-16 | 0.51 | 5.42E-17 | 0.52 | 5.42E-17 | 0.52 | 8.49E-17 | 0.51 | 1.49E-11 | 0.54 | 1.61E-11 | 0.54 | 1.61E-11 | 0.54 | 9.98E-12 | 0.55 |
| 40 ≤ LWP < 50 | 7.86E-13 | 0.61 | 6.51E-13 | 0.62 | 6.51E-13 | 0.62 | 4.37E-13 | 0.62 | 6.43E-10 | 0.64 | 3.63E-10 | 0.64 | 3.63E-10 | 0.64 | 2.53E-10 | 0.65 |
| 50 ≤ LWP < 60 | 2.31E-04 | 0.49 | 8.64E-05 | 0.51 | 8.64E-05 | 0.51 | 5.93E-05 | 0.52 | 2.89E-06 | 0.61 | 2.78E-06 | 0.61 | 2.78E-06 | 0.61 | 7.78E-07 | 0.63 |
| 60 ≤ LWP < 70 | 2.91E-03 | 0.46 | 3.57E-04 | 0.52 | 3.57E-04 | 0.52 | 7.29E-03 | 0.43 | 3.30E-03 | 0.56 | 5.47E-03 | 0.54 | 5.47E-03 | 0.54 | 4.27E-04 | 0.62 |
| 70 ≤ LWP < 80 | 1.40E-03 | 0.59 | 4.34E-04 | 0.62 | 4.34E-04 | 0.62 | 7.04E-04 | 0.61 | 1.65E-03 | 0.62 | 1.20E-03 | 0.63 | 1.20E-03 | 0.63 | 8.28E-04 | 0.64 |
| 80 ≤ LWP < 90 | 1.14E-04 | 0.70 | 3.96E-06 | 0.77 | 3.96E-06 | 0.77 | 9.14E-04 | 0.64 |  |  |  |  |  |  |  |  |

**Supplementary figures**


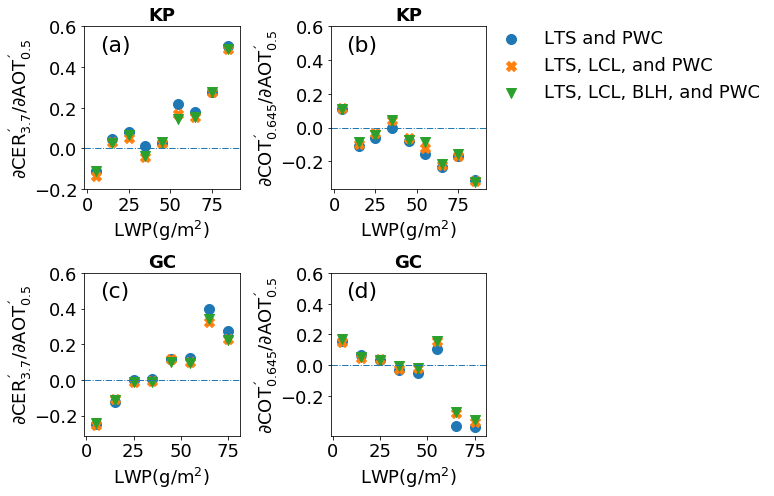


**Figure S1**. Values of ∂$\mathrm{CER}_{3.7}^{´}/{\partial AOT}_{0.5}^{´}$ (left panel) and ∂$\mathrm{COT}_{0.645}^{´}/{\partial AOT}_{0.5}^{´}$ (right panel) for Kanpur (upper panel) and Gandhi College (lower panel) for different sets of metrological factors in multiple linear regression analysis for LWP bins of 10 g/m^2^ spectrum, where $\mathrm{CER}_{3.7}^{´}$, $\mathrm{COT}_{0.645}^{´}$, and $\mathrm{AOT}_{0.5}^{´}$ are normalized anomalies of CER at 3.7 µm, COT at 0.645 µm and AOT at 0.5 µm, respectively. Similarly, LTS, LCL, BLH, and PWC are lower tropospheric stability, lifting condensation level, boundary layer height and precipitable water content, respectively.


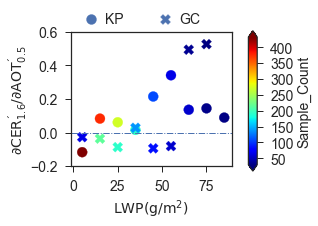


**Figure S2**. Values of $\mathrm{CER}_{1.6}^{´}/\mathrm{AOT}_{0.5}^{´}$ after decoupling the effects of meteorological factors through multiple linear regression for LWP bins of 10 g/m^2^ spectrum for Kanpur (KP) and Gandhi College (GC) sites, where $\mathrm{CER}_{1.6}^{´}$ and $\mathrm{AOT}_{0.5}^{´}$ are normalized anomalies of CER at 1.6 µm and AOT at 0.5 µm, respectively.


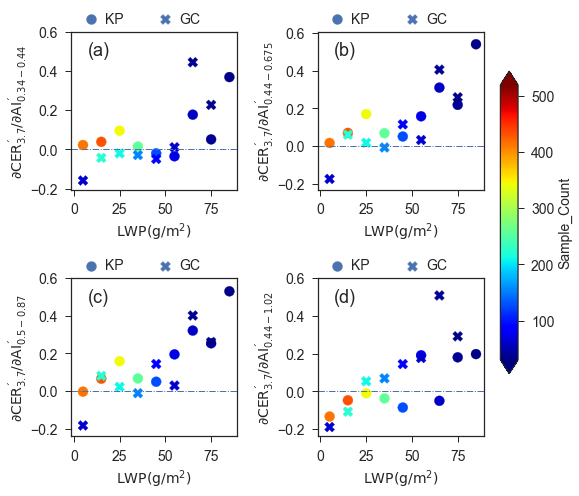


**Figure S3**. Values of $\mathrm{CER}_{3.7}^{´}/\mathrm{AI}_{x}^{´}$ after decoupling the effects of meteorological factors through multiple linear regression for LWP bins of 10 g/m^2^ spectrum for Kanpur (KP) and Gandhi College (GC) sites, where $\mathrm{AI}_{x}^{´}$ is AI determined by multiplying AOT at 0.5 µm with Angstrom exponent (AE) corresponding to wavelengths of **(a)** 0.34, 0.38, and 0.44 µm, **(b)** 0.44, 0.5, and 0.675 µm, **(c)** 0.5, 0.675, and 0.87 µm, and **(d)** 0.44, 0.5, 0.675, 0.87, and 1.02 µm.


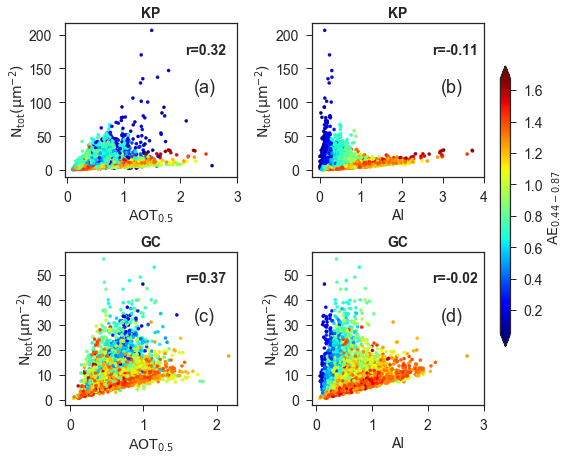


**Figure S4.** Correlations of N_tot_ with **(a)** AOT at 0.5 µm and **(b)** AI (AOT_0.5_×AE_0.44-0.87_) for Kanpur (KP) site, where AOT_0.5_ is AOT at 0.5 µm and AE_0.44-0.87_ is Ångström exponent corresponding to 0.44, 0.5, 0.675, and 0.87 µm. Same in **(c)** and **(d)** for Gandhi College (GC) site.


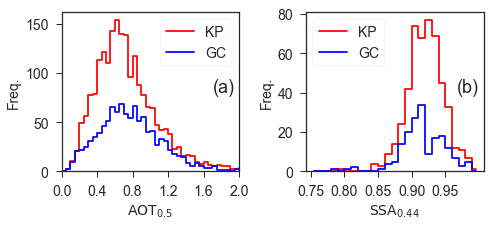


**Figure S5**. Frequency distributions of **(a)** AOT at 0.5 µm µm and **(b)** SSA at 0.44 µm for Kanpur (KP) and Gandhi College (GC) sites.


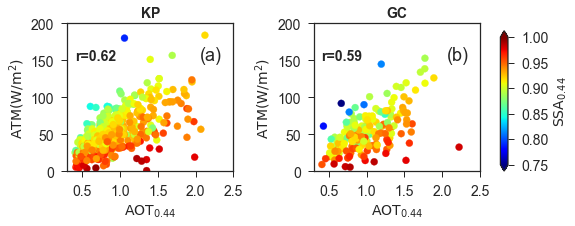


**Figure S6**. Correlation between AOT at 0.44 µm and ATM for **(a)** Kanpur (KP) and **(b)** Gandhi College (GC) sites. The color bar shows SSA at 0.44 µm.
